# Altered Social Cognition in Tourette Syndrome: Nature and Implications

**DOI:** 10.3233/BEN-120298

**Published:** 2013

**Authors:** Clare M. Eddy, Andrea E. Cavanna

**Affiliations:** ^1^Department of NeuropsychiatryThe BarberryNational Centre for Mental HealthBirmingham and School of Clinical and Experimental MedicineCollege of Medical and Dental SciencesUniversity of BirminghamBirminghamUK; ^2^Sobell Department of Motor Neuroscience and Movement DisordersInstitute of Neurology and University College LondonLondonUK

**Keywords:** Tourette syndrome, tics, social cognition, theory of mind, neuropsychology

## Abstract

Behavioural, cognitive and neuroanatomical characteristics of Tourette syndrome (TS) encourage the investigation of social cognitive abilities, which are critical for successful social interaction. This exhaustive review covers studies which have addressed a range of abilities in TS including the understanding of nonliteral language, socially inappropriate exchanges, facial expressions and specific aspects of theory of mind. While the changes in social cognition in TS appear subtle, suitably sensitive measures such as the faux pas task highlight alterations in TS on tasks which involve combinations of emotional information, conflicting perspectives and decision making. Importantly, the differences on social cognitive tasks in TS do not generally reflect a failure to attribute mental states, but rather reflect unconventional responses to social information. Studies have yet to investigate social cognition in children with TS, or evaluate the contribution of common co-morbid disorders. Interpretation of the basis for task deficits is also complex, and research using carefully matched tasks is needed. Nevertheless, it is becoming evident that some aspects of social reasoning involved in decision making are altered in uncomplicated TS, and further investigation in this area may shed light on the mechanisms involved in some of the more socially disabling symptoms associated with this condition.

## 1. Why investigate social cognition in Tourette syndrome?

Social cognition encompasses a collection of skills needed for successful interaction within the social world, including Theory of Mind (ToM): the ability to understand people's mental states, such as beliefs and emotions. As Tourette syndrome (TS) is primarily seen as a movement disorder, it may not initially be apparent why the assessment of social cognition is of value to understanding this condition. However, there are many features of TS which suggest that the study of social cognitive processes is of relevance.

In relation to the behavioural symptoms of TS, Kurlan et al. [1] describe non-obscene socially inappropriate symptoms (NOSIS), which are characterised by urges to act in a socially inappropriate way or make socially inappropriate remarks including insults. These authors report that approximately 25–30% of individuals with TS report experiencing NOSIS. While a range of forms of dangerous complex or self-defeating tics can be seen in TS, NOSIS are specifically related to the surrounding social context, and therefore possess both significant social relevance and impact. The existence of NOSIS phenomena indicate that individuals with TS appear to be sensitive to their social environment and that this may interact in some way with patients' symptoms, prompting the assessment of social cognition.

Cognitive functioning in general has been assessed in TS. A review of studies [2] indicated that individuals without common co-morbidities such as obsessive-compulsive disorder (OCD) or attention deficit/hyper-

ISSN 0953-4180/13/$27.50 © 2013 – IOS Press and the authors. All rights reserved activity disorder (ADHD) exhibit few difficulties, although more demanding tasks, especially those involving inhibition, may yield evidence of impairment. For example, numerous reports [3–6] document inhibitory deficits on the Hayling task [7]. Inhibitory functioning may be critical for ToM. It has been proposed that appreciating another's mental state often requires inhibition of one's own perspective [8], and both developmental and clinical studies have found links between performance on ToM tasks and inhibitory tasks [9,10]. Indeed, the same inhibitory processes recruited during executive tasks contribute to performance on ToM tasks [11] Patients with TS could exhibit poor performance on tasks involving ToM due to inhibitory dysfunction.

Reported striatal abnormalities [12,13] further prompt investigation of social cognition in this patient group. While dysfunction of the basal ganglia in TS are thought to contribute to the occurrence of tics, activation of these subcortical structures can also impact on functioning within cortical brain regions by virtue of connections between frontal brain regions and regions of the striatum [14]. Indeed, recent imaging studies have begun to document evidence of both structural and functional changes in other neural regions which could contribute to social cognitive processes, such as changes in orbitofrontal functioning [15] and amygdala structure [16].

In sum, features of TS relating to behavioural, cognitive and neuroanatomical domains prompt the investigation of social cognitive abilities, which are critical for social interaction and therefore quality of life.

## 2. A review of studies investigating social cognition in TS

To date, there is limited available literature which has investigated aspects of social cognition in TS. Despite this, studies have collectively examined the understanding of nonliteral language, socially inappropriate exchanges, recognition of mental states in facial expressions, specific aspects of theory of mind including the understanding of false belief and reasoning about socially conflicting emotions (Table 1).

An early study assumed social cognitive abilities to be intact in TS and therefore included these patients as a clinical control group [17] when assessing recognition of mental states from photographs of people's eyes (on an early version of the 'Reading the Mind in the Eyes Task': RMET) in participants with autistic spectrum disorder (ASD). The ten patients with TS performed similarly to controls on recognition of facial expressions and Happe's strange stories task [18] and significantly better than individuals with Austistic Spectrum Disorder. However, little information was given about the characteristics of the TS sample.

In 2003, while not explicitly addressing social cognition in TS, Channon and colleagues [3] identified deficits on a problem solving task involving social situations in TS. Participants watched videos of a range of social situations involving predicaments e.g. how to deal with neighbours who have noisy dogs in their flat. For each, participants had to rate ackwardness and generate potential solutions, which were judged according to factors such as social appropriateness and practical effectiveness. Individuals with TS rated the situations as more awkward than the control group. In addition, they tended to generate fewer solutions and selected poorer final solutions.

Shortly afterwards, Channon et al. [4] assessed 15 adult patients with TS and no co-morbid conditions on two tasks involving ToM that contained examples of non-literal language. One was Happe's story task involving examples of double-bluff, persuasion and white lies. ToM is considered to be important for the understanding of non-literal language, as interpretation relies on consideration of the speaker's mental state in addition to the words that are used. No deficits were reported on these tasks. However, this finding may have reflected lack of sensitivity in the measures used, given that later studies began to reveal evidence of changes to ToM in TS.

In a later study, Eddy et al. [6] showed that adults with TS can understand simple false belief according to performance on a story task [19], but exhibit significant difficulty when asked to make judgments about the social appropriateness of behavior during the faux pas task [20]. Patients ($n = 16$) made errors both in failing to recognize faux pas, and in stating faux pas was present in control stories. This differentiation in performance could suggest that emotional elements of social cognition are more likely to lead to altered performance in TS. The finding of a deficit on the faux pas task was particularly interesting in light of the evidence of socially inappropriate behavior in TS. However, patients' errors included both failure to recognize when a socially inappropriate comment had been made, and incorrectly believing offensive remarks had been made on control stories.

The deficit in understanding faux pas was replicated in a new sample of 18 adult patients with TS [21]. This

Table 1
A summary of studies investigating social cognition in Tourette syndrome

| Area of social cognition investigated | Main findings | Implications and directions for future research |
|---|---|---|
| Understanding faux pas | Three studies in different samples have shown that patients with TS reason differently on the faux pas task [6,21,27]. Both omissions and false alarms (thinking there is a socially inappropriate remark when there is not) can occur. | – These findings are particularly interesting given the occurrence of NOSIS in TS. However, NOSIS have yet to be linked to altered performance on ToM tasks<br>– Patients with TS may over-reason about social interactions |
| Problem solving in social situations | One study reported mild deficits in generating and rating solutions aimed at resolving tricky social situations [3]. | – A useful area of research with good relevance to everyday functioning<br>– More exploration of useful tasks is needed in relation to this area |
| Understanding false belief | The understanding of simple false belief appears to be intact in TS, though this has only been investigated in one study [6]. | – TS is not associated with a core deficit in attributing mental states, though social stimuli may sometimes be interpreted in an unconventional way |
| Understanding non-literal language | One study [4] reported understanding of hints, white lies and persuasion seems intact. Another study found sarcasm and metaphorical remarks may be misinterpreted more often (e.g. in a literal sense) in TS [21]. | – This area requires further more systematic study<br>– It is important to consider task requirements and screen for language problems |
| Interpretation of complex mental states from photographs of eyes | Patients with TS may show small differences in the interpretation of facial expressions [23]. | – Studies have yet to report performance on basic facial expression recognition measures<br>– These tasks provide a useful complementary approach to verbal tasks |
| Reasoning involving socially competitive emotions | In one study, TS was associated with difficulties on a facial expression task involving reasoning about envy and gloating [23].<br>Patients with TS may respond differently to unfair offers in the ultimatum game [23]. | – Stimuli involving conflicting emotions seem to be more likely to elicit unconventional reactions in TS<br>– The potential influence of emotion on decision making is an important area of research |
| Interpretation of humorous stimuli | Patients with TS may exhibit unconventional interpretations of material with humorous content [23]. Understanding of non-literal language may contribute. | – This is a complex area of research and only one study has been conducted so far<br>– Humour and creativity seem relevant to some kinds of complex verbal tics (e.g. NOSIS) |

study further demonstrated impairments in understanding examples of non-literal language in TS, such as sarcasm and metaphor. When patients with co-morbidities were removed from analysis, impairments were present in the subgroup of uncomplicated patients ($n = 11$). The non-literal language task which revealed evidence of impairment was the Pragmatic Story Comprehension Task (PSCT) [22]. A task requiring the interpretation of hints was also included, and patients with TS performed normally on this task. Performance may have differed across the non-literal language tasks because while the hinting task simply required participants to interpret a remark, the PSCT was likely to be more sensitive as it required participants to first decide which comments made sense from a selection of appropriate metaphorical, literal and sarcastic remarks and a nonsense statement. Patients with TS were most likely to decide that sarcastic remarks (and then metaphorical remarks) did not make sense, but performed well with literally appropriate remarks and nonsense statements, making a general language deficit a poor explanation for performance. Of further interest was a correlation between performance on the PCST and time measures on a black and white stroop task, which could indicate problems with non-literal language were linked to inhibitory problems. For example, patients could sometimes have found it hard to inhibit the literal meaning of a sarcastic remark in order to understand it's appropriateness in the story context.

A third study conducted by Eddy and colleagues [23] tested the performance of eighteen patients with TS on four tasks involving ToM. The rationale for task selection was also based on the likely involvement of ventromedial prefrontal regions in task performance, as this region is thought to be important for the understanding of faux pas and sarcasm. One task was the Reading the Mind in the Eyes task (a later version [24] than that used by [17]), and another was a task which involved selecting facial expressions based on matching and contrasting emotions (gloating and envy) in the Socially Competitive Emotions Task (SCET) [25]. Patients also completed a multiple choice task which involved understanding the joke in humorous cartoons (i.e. thinking about a cartoonist's intentions) devised by the authors, and a version of the Ultimatum game, an economic decision making task linked to ToM. Patients with TS exhibited significant deficits on all four tasks in comparison to a matched control group. Furthermore, ToM deficits were present in uncomplicated TS ($n = 13$). It was suggested that these changes could reflect dysfunction within frontostriatal pathways involving ventromedial prefrontal cortex (VMPFC) and the amygdala in the pathophysiology of TS. Indeed, one recent study did report altered amygdala activation in TS in relation to social stimuli [26].

A more recent study has provided further support for the reported deficit in understanding faux pas in TS [27] in a group of 20 uncomplicated patients. This study also investigated the regulation of autobiographical emotional self-disclosures, but found no overall differences in comparison to controls for this measure.

## 3. Summary of study findings: Nature of changes in social cognition in TS

### *3.1. How pervasive are differences in social cognition in TS?*

While not all patients perform differently to controls, studies indicate that changes to ToM are common in uncomplicated TS, while no study has systematically examined the potential contribution of co-morbid conditions such as OCD or ADHD to performance on social cognitive tasks. As yet, it has not been established whether any specific clinical characteristics are related to social cognitive alterations in TS.

Given the variation in findings on different instruments across studies, some of the changes in social cognition in TS may be rather subtle, and only evident on certain sensitive measures. It seems that those measures which involve combinations of emotional information, conflicting perspectives and decision making or problem solving (e.g. the faux pas task) may show most differences in TS.

### *3.2. What is the precise nature of changes in social cognition in TS?*

Importantly, the differences on social cognitive tasks in TS do not generally reflect a failure to attribute mental states as may be seen in ASD, but more often imply different responses to or interpretation of, social information. For example, on some occasions, patients signal a socially inappropriate remark may have been made in a story when it had not, or mistakenly interpret that the protagonist's offensive remark is deliberate [6, 21]. Such errors indicate that differences are apparent in the way that patients reason about social interaction and make attributions about the motives and intentions behind social behaviour.

It could be that individuals with TS exhibit increased emotional reactivity when tasks highlight conflict within social interaction, such as situations where one person may be negatively affected by another person's actions. This could help explain why patients with TS were more likely to reject offers of money during the Ultimatum Game [23], where an unfair proposer offered them an ultimatum choice of an unfair share of money or nothing. Indeed, this could also explain the heightened ratings of awkwardness in response to a social predicaments task exhibited by patients with TS in comparison to controls reported by Channon et al. [4]. Increased emotional reactivity in response to social stimuli associated with negative emotion could lead to reduced 'logical' control over decision making.

### *3.3. Do the differences on social cognitive tasks represent changes to ToM per se?*

Patients with uncomplicated TS (without co-morbid behavioural problems) exhibit inhibitory deficits and it has been suggested that appreciating another's mental state often requires inhibition of one's own perspective [8]. Indeed, the same inhibitory processes recruited during executive tasks can contribute to performance on ToM tasks [11]. The social cognitive alterations revealed in the reviewed studies often appeared independent of executive dysfunction. For example, while patients with TS exhibited mild inhibitory impairments on the Hayling task [6], a black and white Stroop [21] and on a verbal fluency measure [23], no significant correlations were found between executive and ToM measures. Having said this, the administered executive tasks may not have been sensitive enough to the exact executive skills relevant to the accompanying social cognitive tasks. Further research is needed to establish whether such changes are completely independent of subtle or selective difficulties with aspects of executive function.

One complicating factor is that executive functions can make incidental (e.g. working memory processes involved in remembering story events) or more fundamental (e.g. inhibition needed to ignore one's own viewpoint) contributions to performance on social cognitive tasks. While incidental contributions can be limited to some degree, it is likely to be impossible to disentangle executive skills which are integral to the perspective taking process.

### 3.4. *What limitations are associated with the available evidence?*

There are numerous limitations associated with the reviewed studies which encourage further research. These mainly concern methodological issues related to the selection of samples and materials.

At the time of this review, no studies were available which had investigated social cognition in children with TS. This would provide a critical opportunity to determine whether TS may lead to developmental changes in the acquisition of abilities related to ToM. In addition, studies have not assessed the likely contribution of common co-morbid disorders such as OCD or ADHD to social cognitive performance. It may be speculated that these conditions will exert different impacts. For example, the attention deficits associated with ADHD could lead to more failures to attribute mental states whereas OCD could be associated with increased rumination about the perspectives of others.

It is often difficult to determine the basis for patients' difficulties on tasks. For example, general language or concentration problems could influence performance on social cognitive tasks. Therefore there is a need for better controlled studies with matched tasks with similar structure containing social/emotional versus non-social/emotional material. Comparison of verbal and non-verbal measures, and measures focused on more cognitive (e.g. beliefs) versus affective (e.g. emotions) aspects of social cognition could also offer much needed insight.

Finally, but importantly, is also hard to see how difficulties with social cognitive studies in the lab may be reflected in everyday social behaviour. One study [27] attempted to address this point, but further studies are needed.

## 4. Implications of changes to social cognition in TS

### 4.1. *Implications for behavioural and clinical characteristics*

Aspects of social reasoning involved in problem solving and decision making seem to be vulnerable to impairment in TS. It is when the social/emotional info needs to be applied in some adaptive way which seems to be the problem, or when the task involves multiple perspectives which conflict (as in the faux pas task and SCET). Patients' performance on social cognitive tasks could be affected by sensitivity or insensitivity to social information or emotive cues, which could result in abnormal interpretations and poor decision making. Similar mechanisms may apply to NOSIS. Certainly in the case of NOSIS there are emotional elements, multiple people's perspectives and context dependent adaptive behaviour is required.

One way social cognitive alterations in TS could be related to the occurrence of NOSIS is through poor inferential reasoning about people's mental states in response to their behaviour. For example, some patients could make socially inappropriate remarks because they fail to consider other people's feelings. However, many patients with NOSIS do seem to be aware of the inappropriate nature of the remarks they make, and original explanations for NOSIS therefore suggested these symptoms resulted from an inhibitory problem [1]. However, such as explanation is limited in that it does not explain where the urge (which is difficult to inhibit) arises from in the first place.

Here the author proposes an alternative model for NOSIS which explain how these urges and behaviours could occur in patients who are aware of the negative affective consequences of such socially inappropriate behaviours. There are key roles for emotional dysregulation and autonomic reactivity in this model. Some mechanisms described in the model could be applied to help explain altered performance on social cognitive tasks in TS.

The first stage of NOSIS generation involves environmental evaluation, or the patient becoming aware of their social surroundings. NOSIS are dependent on the stimuli in the patient's immediate environment. In patients who exhibit these symptoms, an initial evaluation of socially related reinforcement contingencies signalled by the environment could be associated with a greater awareness of potential behaviours linked to negative affective consequences (e.g. shouting "fatty" on seeing a person who is overweight). This process is likely to involve the orbitofrontal cortex which is involved in the appraisal of stimuli in relation to associated reinforcement contingencies [28].

An awareness of the negative connotations associated with a particular behaviour may be associated with a change in autonomic arousal. Changes in autonomic arousal could act as a deterrent to warn against behaviour linked to negative reinforcement. Autonomic arousal is regulated by the heavily interconnected amygdala and insula. These neural structures process negative affective experiences and contribute to social judgments [29]. The anticipatory autonomic arousal associated with the awareness of potential socially inappropriate behaviours could be heightened in TS.

In comparison to individuals who do not experience NOSIS, those with TS who do could find this arousal leads to greater physiological and/or psychological discomfort. This increased discomfort could lead to an urge to release the behaviour in order to reduce the anticipatory arousal. Feeling an urge to act and feeling discomfort as a result of the increased arousal are likely to involve the insula and anterior cingulate cortex (ACC). The insula both senses interoceptive information and is involved in the generation of subjective feelings and urges [30]. The ACC is also activated by autonomic arousal [31]. In healthy individuals, ventromedial prefrontal regions help regulate arousal and somatic state through the amygdala [32]. In TS, failure of cortical regions (such as the VMPFC and\or ACC) to control or regulate this arousal could lead to NOSIS.

This model would predict that efficacious treatments for NOSIS could either help reduce or attenuate relevant socially elicited autonomic arousal, or enhance cortical control over the arousal.

### *4.2. Implications for neurobiological alterations in TS*

Reported alterations in performance on social cognitive tasks in TS may implicate changes in the structure and functioning of numerous brain regions including medial and lateral orbitofrontal cortex, anterior cingulate and the amygdala.

If patients' deficits on ToM tasks do reflect changes in ToM *per se*, this may suggest activation changes within frontostriatal pathways involving VMPFC, as dysfunction of this region can impair performance on the faux pas task [33], reasoning about socially competitive emotions [34] and decision making during the ultimatum game [35].

Patients with TS exhibit some characteristics that are similar to those displayed by patients with medial orbitofrontal dysfunction. For example, patients with TS exhibit NOSIS whilst inappropriate social behaviour is exhibited by patients with sustained orbitofrontal damage [36]. Dysfunction of this region has also been linked to poor prioritization of solutions to interpersonal problems [37]. Changes in OFC activation in TS were reported by Braun et al. [15].

Ventromedial prefrontal regions are also heavily connected to the amygdala. Dysfunction of this region may affect performance on social cognitive tasks features in the reviewed studies such as the faux pas task [38] and UG [35]. Indeed, recent studies have reported alterations to the amygdala in TS [39–41] including altered functional connectivity involving this region [42].

In terms of frontostriatal circuitry, dysfunction within the ACC circuit, which involves the ventral striatum [14] is likely to have contributed to the differences in performance on ToM tasks that were demonstrated by patients with TS. The suggestion that the ACC is likely to be dysfunctional in TS is supported by imaging and behavioural evidence. As mentioned earlier, patients with TS exhibit mild deficits on the Hayling task [7], a test which is sensitive to ACC function [43]. Imaging studies have revealed abnormalities in this region in TS, including evidence of decreased grey and white matter [44]. Thus, increased ACC activity occurs during tic suppression [45] and there is a positive correlation between activity in this region and tic severity [46]. Engagement of the ACC frontostriatal circuit in tic suppression may result in fewer resources being available for social cognitive tasks. However, no relationships have been reported between social cognitive performance and tic severity as yet.

Problems with inhibition in TS could implicate the ACC or inferior frontal gyrus (IFG). Therefore, if difficulties with social cognitive abilities are linked to inhibitory dysfunction, these regions will be relevant. Dysfunction within the lateral orbitofrontal cortex circuit could also be implicated in patients' deficits on tasks involving non-literal language. The IFG, which can be considered part of the lateral orbitofrontal circuit, is active during the processing of sarcasm [47]. Studies have reported abnormalities of the IFG in patients with TS. For example, one imaging study reported decreased white matter in this region [44]. Changes in the functioning of the lateral orbitofrontal region were also found by Jeffries et al. [48]. The IFG is also active during the Hayling task [43] and some studies implicate this structure in inhibitory control [49]. It may therefore be speculated that dysfunction of the IFG in TS could lead to difficulties in the comprehension of non-literal language.

## 5. Conclusion

A review of study findings indicates that TS can be associated with changes in reasoning on tasks involving social and emotional elements. While there is enough evidence to say ToM can be different in TS, further study of social cognition in TS is clearly merited in order to determine whether these differences are linked to psychosocial factors, specific cognitive alterations, emotional dysregulation or neurological changes. There is a need for the application of specially designed batteries of matched tasks to allow better specification of any identified performance differences. Future studies should also assess spontaneous aspects of social cognition in addition to cued performance, in both adults and children, while seeking to identify relationships between task performance and factors such as symptom severity and autonomic reactivity. Investigation of the social and emotional changes linked to TS provides a valuable opportunity to both better understand the global impact of this condition and address factors highly relevant to daily functioning and quality of life.
